# Identification and validation of tumor-specific T cell receptors from tumor infiltrating lymphocytes using tumor organoid co-cultures

**DOI:** 10.1007/s00262-024-03749-8

**Published:** 2024-07-02

**Authors:** Zhilang Li, Lisha Ma, Zhaoya Gao, Xiya Wang, Xuan Che, Pengchong Zhang, Yixian Li, Qianjing Zhang, Tianxing Liu, Yuan Sun, Yun Bai, Hongkui Deng

**Affiliations:** 1 Department of Cell Biology, School of Basic Medical Sciences, Peking University Health Science Center, Beijing, 100191 China; 2 Department of Gastrointestinal Surgery, Peking University Shougang Hospital, Beijing, 100041 China; 3 Peking-Tsinghua Center for Life Sciences, Peking University, Beijing, 100091 China

**Keywords:** Patient derived organoids, Tumor-infiltrating lymphocytes, T cell receptor-engineered T cells, Colorectal cancer, Immunotherapy

## Abstract

T cell receptor-engineered T cells (TCR-Ts) therapy is promising for cancer immunotherapy. Most studies have focused on identifying tumor-specific T cell receptors (TCRs) through predicted tumor neoantigens. However, current algorithms for predicting tumor neoantigens are unreliable and many neoantigens are derived from non-coding regions. Thus, the technological platform for identifying tumor-specific TCRs using natural antigens expressed on tumor cells is urgently needed. In this study, tumor organoids-enriched tumor infiltrating lymphocytes (oeT) were obtained by repeatedly stimulation of autologous patient-derived organoids (PDO) in vitro. The oeT cells specifically responded to autologous tumor PDO by detecting CD137 expression and the secretion of IFN-γ using enzyme-linked immunospot assay. The measurement of oeT cell-mediated killing of three-dimensional organoids was conducted using a caspase3/7 flow cytometry assay kit. Subsequently, tumor-specific T cells were isolated based on CD137 expression and their TCRs were identified through single-cell RT-PCR analysis. The specificity cytotoxicity of TCRs were confirmed by transferring to primary peripheral blood T cells. The co-culture system proved highly effective in generating CD8^+^ tumor-specific oeT cells. These oeT cells effectively induced IFN-γ secretion and exhibited specificity in killing autologous tumor organoids, while not eliciting a cytotoxic response against normal organoids. The analysis conducted by TCRs revealed a significant expansion of T cells within a specific subset of TCRs. Subsequently, the TCRs were cloned and transferred to peripheral blood T cells generation engineered TCR-Ts, which adequately recognized and killed tumor cell in a patient-specific manner. The co-culture system provided an approach to generate tumor-specific TCRs from tumor-infiltrating lymphocytes of patients with colorectal cancer, and tumor-specific TCRs can potentially be used for personalized TCR-T therapy.

**Supplementary Information:**

The online version contains supplementary material available at 10.1007/s00262-024-03749-8.

## Introduction

With the developing landscape of cancer treatment, immunotherapy has become a major component in the management of multiple cancers. Empirical evidence has demonstrated the efficacy of T cell receptor-engineered T cell therapy in inducing potent anti-tumor responses in several solid tumors through adoptive cell transfer (ACT) [1–4]. These clinically effective anti-tumor responses are mediate by tumor-specific TCRs recognition of tumor antigens. However, the major bottleneck in the wide application of TCR-T therapy is how to efficiently and accurately isolate tumor-specific TCRs. Most studies have conducted comprehensive tumor-specific TCRs screening by incubating antigen presenting cells (APCs) with T cells, which relied on either loading synthetic neoantigen peptides onto APCs or electroporating tandem minigenes encoding tumor neoantigen into APCs to generate tumor cell surrogates [5, 6]. These methods highly depends on the prediction and using appropriate tumor antigens, while current prediction algorithms for tumor neoantigens are unreliable and some neoantigen peptides can’t be a naturally presented epitope [7, 8]. Furthermore, only a limited number of neoantigens have the potential to elicit an immune response in epithelial cancer [9], and many tumor neoantigens were derived from non-coding regions [10]. Currently, there is an urgent need to find more sources of tumor antigens.

Tumor organoids presenting various natural tumor neoantigens could be used in an unbiased manner to generate tumor-specific T cells [11, 12]. Tumor organoids preserve histopathologic features and genomic characteristics of the original tumor, including individual cancer mutations and mutation burden [13–15]. Previous studies have demonstrated that colorectal tumor organoids displayed diverse HLA peptide presentation, including tumor-specific antigens, that renders organoids a suitable model for the generation of natural tumor antigens for stimulating the expansion of tumor-specific T cells in vitro [16].

In this study, tumor organoids are used as the source of antigens to address the issues of imprecise antigen prediction and time-consuming tumor antigen synthesis. As well as for the source of TCR repertoire, we choose autologous tumor-infiltrating lymphocytes (TILs), due to TILs have greatly enriched for tumor specific T cells and are optimal for the identification of tumor-specific TCRs [17–20]. In addition, enriching tumor-specific T cells in vitro can avoid immunosuppressive interference in the tumor microenvironment through culture optimization.

Here, we developed a co-culture system of tumor-specific T cells that can be efficiently expanded by co-culturing tumor organoids with autologous TILs, and subsequently their TCRs were isolated and transferred to peripheral T cells to generate tumor-specific TCR-Ts. The capacity of TCR-T cells to exhibit tumor-specific reactivity and cytotoxicity against autologous tumor cells have been observed and evaluated. This strategy provides an approach to identify tumor-specific TCRs in vitro rapidly, which can be a potential strategy for future clinical application of personalized TCR-T therapy.

## Materials and methods

### Patient samples

Tumor tissues and peripheral blood were obtained from patients with a confirmed diagnosis of colorectal cancer. The fresh surgically resected tumor samples were placed in sterile 15 mL tubes containing Advanced DMEM/F12 (GIBCO) supplemented with pencillin/streptomycin (GIBCO), subsequently divided into multiple fragments for TILs culture and generation of patient derived organoids (PDO). This study was approved by the Medical Ethical Committee of the Peking University Shougang Hospital and written informed consent was obtained from all patients.

### PDO and TILs establishment

The establishment of PDO was performed in accordance with the previously described method [12]. Briefly, the fresh tumor tissue and normal colon tissue was washed with ice-cold phosphate-buffered saline (PBS) buffer and minced into small fragments. The fragments were enzymatically digested using 10 μg/mL hyaluronidase type IV (Sigma-Aldrich), 1.5 mg/mL collagenase II (Sigma-Aldrich), and 10 μM Y-27632 (Sigma-Aldrich) for approximately 45 min before embedding in Matrigel (Corning). Tumor organoids were cultured in Advanced DMEM/F12 (Gibco) supplemented with 10 mM HEPES (Gibco), 1 × B27 supplement without vitamin A (GIBCO), 1 × N2 (Gibco), 2 mM Ultra-glutamine I (Lonza), 500 ng/ml R-Spondin-1(Peprotech), 100 ng/ml Noggin (Peprotech), 10 nM prostaglandin E2 (Cayman Chemicals), 3 μM SB202190 (Cayman Chemicals), 10 nM Gastrin I (Sigma-Aldrich), 50 ng/ml human recombinant EGF (Peprotech), 0.5 μM A83-01(Tocris), 10 mM Nicotinamide (Sigma-Aldrich), 1 mM N-acetylcysteine (Sigma-Aldrich), 1 × Pencillin/Streptomycin (Gibco) and 1 × Primocin (Invivogen), 10 μM Y-27632 supplemented with medium for the first 3 days. Normal colon organoid medium is identical to tumor organoid medium, except that it needs to be supplemented with 50% Wnt3a-conditioned medium. The Wnt3a-conditioned medium were obtained from L-Wnt3a cells. PDO was passaged approximately every 7–10 days using TrypLE Express (GIBCO) for about 10 min at 37 ℃ to dissociate the PDO into single cells. After dissociation, the cells were embedded in Matrigel (Corning, USA), and the medium was changed every 3–4 days.

TILs were generated as previous described [21]. Briefly, the tumor samples were minced into approximately 1–2 mm fragments after washed with ice-cold PBS buffer, and then put in a digestion medium consisting of 1 mg/ml collagenase (Sigma-Aldrich, USA) and 30U/ml DNase (Sigma, USA) at 37 °C for about 60 min. Following digestion, the cells were seeded into a 6-well plate containing T cell media supplemented with X-VIVO 15 (Lonza, USA), 2 mM Glutamax (Life Technologies, USA), 6000 U/mL IL-2 (Perprotech, USA), 10 ng/mL IL-15 (Perprotech, USA), 10 ng/mL IL-21 (Perprotech, USA), 1 × Primocin (Invivogen) and 30 ng/mL anti-human CD3 antibody (OKT-3, Miltenyi, Germany). TILs were cultured at 37 °C with 5% CO2 and passaged until enough TILs were available for screening tumor-specific T cells.

### Peripheral blood lymphocytes

PBLs were obtained from freshly peripheral blood through Ficoll Paque plus (GE, DN25) density gradient centrifugation. Subsequently, the isolated PBLs were cryopreserved in liquid nitrogen for future utilization.

### Histological analysis and immunohistochemistry staining of PDO

Organoids were collected (e.g., 6-well plate, collection of 1–2 well organoids, about 300–800 organoids) and subsequently fixed in 4% paraformaldehyde for 30 min at 4 °C, followed by gradient dehydration with ethanol and embedding in paraffin wax. The paraffin blocks were cut at a thickness of 5 μm using a paraffin microtome (Leica, RM2245) and subjected to routine hematoxylin–eosin (H&E) staining.

For immunohistochemistry staining, paraffin sections were routinely deparaffinized and incubated for 30 min in 3% H_2_O_2_ to inactivate endogenous peroxidase. Heat-induced antigen retrieval was performed using citric acid for 15 min at 95 °C. The sections were incubated with primary antibodies overnight at 4 °C. Then the HRP-conjugated second antibodies were stained for 1 h at room temperature (Supplementary Table S1). The bound antibody was visualized using the DAB detection kit (ZSGB-BIO, ZLI-9019) and counterstained with hematoxylin. The results were recorded using a light microscope (Olympus).

For immunofluorescent analysis, the organoids were fixed in 4% paraformaldehyde (DingGuo) for 15 min at 4 °C. Subsequently, they were permeabilized with 0.1% Triton X-100 (Sigma-Aldrich). Then the organoids were incubated with primary antibodies overnight at 4 °C, followed by incubation with secondary antibodies for 1 h (Supplementary Table S1). Nuclear staining was performed using 4',6-diamidino-2-phenylindole dihydrochloride (DAPI; Sigma). The resulting images were captured using a confocal microscope (Zeiss, LSM780).

### TILs and PBLs phenotypic characterization analysis

For CD39^+^CD103^+^ positive T cell analysis, TILs and PBLs from CRC patients were stained with anti-human CD3-FITC, anti-human CD8-PE, anti-human CD39-PE-Cy7, anti-human CD103-APC antibodies cocktails and 7AAD (Invitrogen). For anti-human CD8^+^PD1^+^ positive T cell analysis, TILs and PBLs were stained with anti-human CD3-FITC, anti-human CD8-PerCP5.5, anti-human CXCL13-PE antibodies cocktails and 7AAD. For CD8^+^PD1^+^ positive T cell analysis, TILs and PBLs were stained with anti-human CD3-FITC, anti-human CD8-PerCP5.5, anti-human PD-1-PE antibody cocktails and 7AAD (supplementary Table S2). Then samples were analyzed by gating of CD3^+^CD8^+^ T cells of all flow cytometry, the data were analyzed using FlowJo software.

### Co-culture of tumor organoids and autologous TILs to generate tumor organoid-enriched T cells

Co-culture of single cell-derived organoids and autologous TILs were performed to enrich tumor-specific T cells. Briefly, two days before co-culture, tumor organoids embedded in Matrigel were stimulated with decitabine (DAC) cocktails (10 μM DAC (Sigma Aldrich), 100 U/mL IFNγ (ACRO) and 10 ng/mL TNF-α (ACRO)) for 48 h. Previous studies revealed that DAC cocktails can increase the expression of HLA by either suppressing DNA methylation or promoting the expression of HLA-associated TAP or LMP genes [22–24].

Next, the tumor organoids were dissociated into single cells using TrypLE Express and then resuspended in T cell medium. Prior to the experiment, a 96-well U-bottom plate was prepared by coating with 5 µg/mL anti-CD28 antibody in PBS (50 µL per well) and subsequently wrapping in parafilm overnight at 4 °C. The anti-CD28-coated plates were then washed twice with PBS. TILs were seeded at a concentration of 10^5^ cells per well and co-cultured with autologous single cell organoids at a concentration of 10^4^ cells per well at a 10:1 effector: target ratio. Co-cultures were performed in T cell medium (X-VIVO15 medium supplemented with 10% human AB serum, 2 mM glutamine, 150 U/ml IL-2, 10 ng/mL IL-15, 10 ng/mL IL-21, 1% penicillin–streptomycin, 20 μg/mL anti-PD-1 blocking antibody (Biolegend)) and cultured at 37 °C with 5% CO2. On the 7th day, co-cultures TILs were re-stimulated with fresh organoids at a ratio of 10:1 for an additional 7 days. The medium was half-changed every 1–2 days, and co-culture ended on day 14. TILs without tumor organoids stimulation were set up as a control.

### Three-dimensional (3D) organoid killing assays

Organoids killing assay was performed in accordance to previously described with slight modification [25]. In brief, tumor organoids were stimulated with DAC cocktails for 48 h, and part of the organoids were dissociated into single cells and then counted to infer the number of tumor cells at a effector: target = 10:1 ratio. 1 mM of (Invitrogen) celltrace yellow cell proliferation kit (Invitrogen, cat. no. C34567) was used to stain organoids for 20 min. 3D tumor organoids and normal organoids were resuspended in T cell medium, and seeded in flat-bottom 96-well plate with 1 × 10^5^ autologous oeT cells obtained by 14 days of tumor organoid co-culture. Green-fluorescent caspase 3/7 probe (Invitrogen, cat. no.C10723) was added during co-culture at 1:2000 dilution to visualize tumor cells undergoing apoptosis. After 20 h of co-culture, part of the cells (about 3 wells) were observed and photographed using fluorescence microscope, and part of the cells (about 6 wells) were used for flow detection of apoptotic cells. For MHC I/II blocking assay, tumor organoids were pretreated with 50 μg/mL pan-MHC I antibody (Biolegend, 311428) and pan-MHC II antibody (BD, 555556) blocking for 40 min before co-culture. Part of organoids dissociated into single cells with TrypLE Express, and the cells stained with anti-human CD3-FITC (Biolegend), anti-human CD8-PE (Biolegend), anti-human CD107a-PE-Cy7 (Biolegend) and 7AAD (supplementary Table S2). Then the samples were analyzed by flow cytometry.

### Isolation of tumor-specific T cells after repeated stimulation with autologous tumor cells

To evaluate tumor reactivity of oeT cells, oeT cells were collected after 14 days of incubation with tumor organoids and resuspended in T cell medium. The oeT cells were then restimulated with tumor organoids and normal organoids at a 1:1 effector: target ratio, respectively. Subsequently, seeded in anti-CD28-coated plates in the presence of 20 mg/mL anti-PD-1 and co-cultured for 24 h. TILs without organoids stimulation and normal organoids stimulation were set up as controls. Cells were washed twice in PBS and stained with the following antibodies: anti-human CD3-FITC (Biolegend), anti-human CD8-PE (Biolegend), anti-human CD137-APC (Biolegend) and 7AAD for 30 min at 4 °C (Supplementary Table S2). Then the cells were washed twice with PBS. The expression of surface markers were quantified, and the CD8^+^CD137^+^ positive T cells were sorted into 96-well PCR plates using the Symphony S6 (BD) and analyzed using FlowJo software. Subsequently, the 96-well PCR plates were immediately stored at −80 °C.

### T cell receptor sequencing and analysis

Single-cell RT-PCR is based on a previously described method [26]. The PCR primer sequences are listed in supplementary Table S3. Briefly, the first round of RT-PCR produces single-cell cDNA. The reaction conditions for the first-round RT-PCR were as follows: the reverse transcription reaction for 30 min at 50 °C; then 95 °C for 15 min and 30 cycles of 94℃ for 30 s, 52 °C for 30 s, 72 °C for 1 min; 72 °C for 10 min. Subsequently, RT-PCR cDNA from the first round was used as a template for the second round of TCRα/β PCR. The reaction conditions for the second round PCR are as follows: 98 °C for 1 min and 43 cycles of 98 °C for 10 s, 52 °C for 10 s, 72 °C for 45 s; 72 °C for 10 min. The PCR products were sequenced after gel cutting and purification. The TCR sequences were analyzed using the IMGT/V-Quest tool (http://www.imgt.org/).

#### TCR construction and transduction of PBLs

Construction of retroviral vectors encoding TCRs was used the methods as described previously [5, 27]. Briefly, we constructed the TCRs in a β chain-α chain order, fusing the TCRβ V-D-J of the Vβ regions to the mouse TCRβ constant chain and the TCRα V-J regions to the mouse TCRα-constant chain. As previously described, the mouse constant region was modified with a disulfide bond and a hydrophobic substitution [28–30]. Additionally, furin-SGSG-P2A sequence was introduced between the TCRβ chain and TCRα chain. The TCR sequences were synthesized after codon optimization (GenScript) and subsequently inserted into the lentivirus vector.

TCR transduction was performed in accordance to previously described with slight modification [22, 31]. In brief, human PBLs from healthy or autologous donors were stimulated with 50 ng/ml OKT3 in T cell media for 48 h before transduction. HEK293T cells were transfected with the lentivirus plasmid encoding TCRs and the envelope-encoding plasmid using CaCl2 to produce retroviral supernatants. After 48 h, the viral supernatants were harvested and then used to infect PBLs in the presence of polybrene (8 μg/mL). 3 days after infection, TCR-positive T cells were isolated through flow cytometry using a mouse TCR beta monoclonal antibody (Invitrogen, 17–5961-82). The sorted cells were subsequently confirmed using anti-human CD3-FITC (Biolegend, USA), anti-human CD8-PE (Biolegend, USA) and mouse TCR beta monoclonal antibody (eBioscience, USA).

#### Enzyme-linked immunospot assay (ELISPOT)

The human IFN-γ ELISPOT assay was performed according to the instructions provided by the manufacturer of the commercially available human IFN-γ ELISPOT kit (Dakewe, cat. No. 2110005, China). Briefly, before co-culturing, T cells rested in cytokine-free medium for 24 h and tumor organoids were treated with DAC cocktails for 48 h. A total of 1 × 10^4^ T cells and 1 × 10^4^ autologous organoids were incubated at 37 °C with 5% CO2 for 20 h in cytokine-free medium. Phytohemaggluinin (PHA) was added as a positive control, and medium was added as a negative control. The Immunospot plate reader (Cellular Technologies, USA) was used to analyze the number of IFN-γ positive spots.

#### Enzyme-linked immunosorbent (ELISA) assay

Human IFN-γ cytokine release was quantified using the human ELISA kit (Dakewe Biotech cat. no. 1110002, China). Briefly, T cells were rested in cytokine-free medium for 24 h, and organoids were stimulated with DAC cocktails for 48 h prior to co-culture. A total of 5 × 10^4^ T cells and 5 × 10^4^ autologous organoids were incubated together for 24 h in cytokine-free medium, and then the co-cultured supernatant was collected. IFN-γ cytokine secretion was measured using a commercially available human IFN-γ ELISA kit, following the manufacturer's protocols. In the MHC I blocking experiment, tumor organoids were pretreated with 50 μg/mL of pan-MHC I antibody (Biolegend, 311428) for 40 min before co-culture. The tumor recognition was tested in technical replicate experiments.

#### Statistics analysis

Data statistical analysis was performed using GraphPad Prism 7.0. The statistical analysis was conducted with a student's *t*-test. *p*-values < 0.05 were considered statistically significant, denoted by *(*p* < 0.05), **(*p* < 0.01), and ***(*p* < 0.001). All experiments were conducted more than three times independently.

## Results

### Tumor organoids retained the characterization of parent tumor tissue

**Fig. 1:**
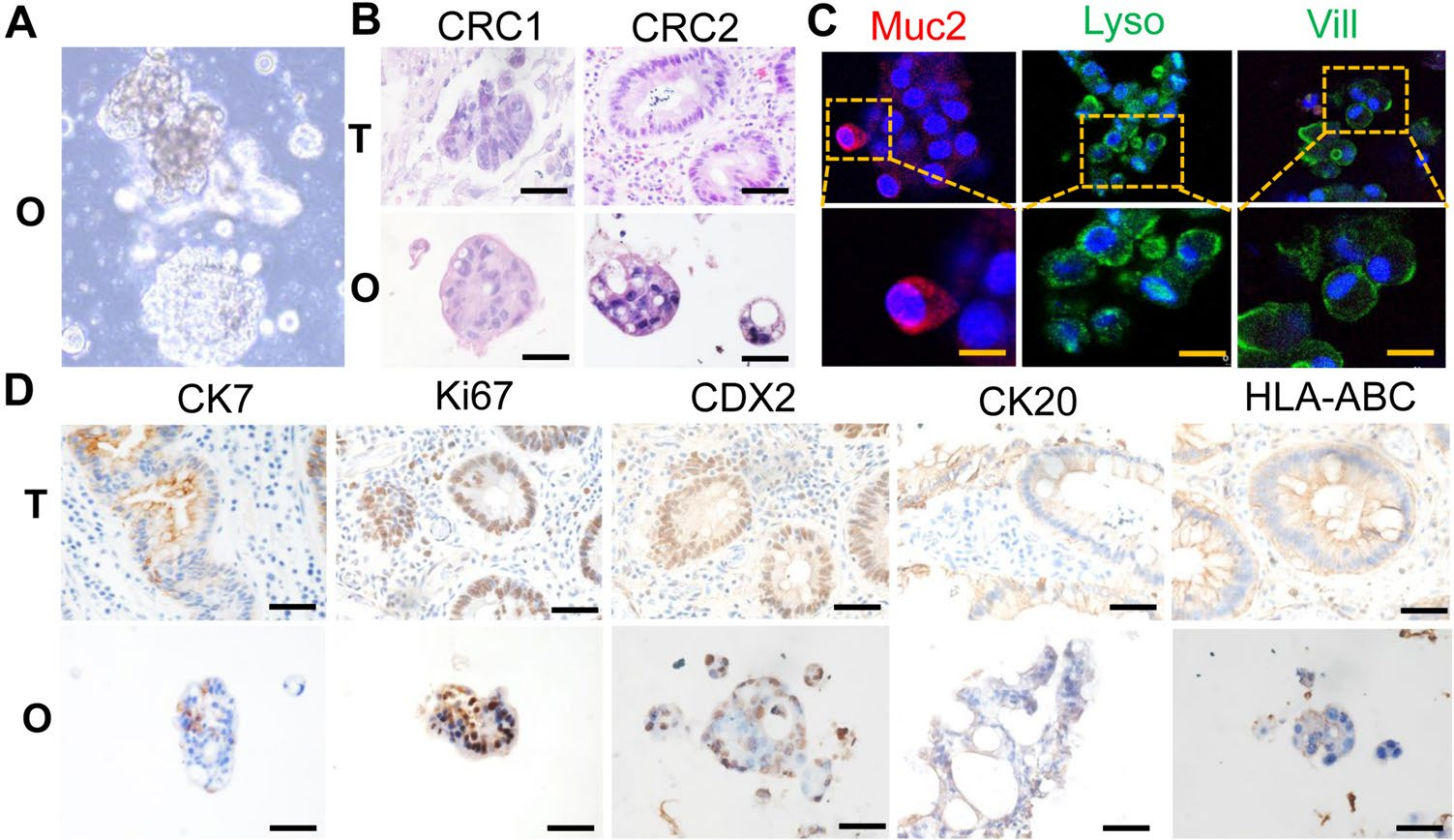
Histological analysis revealed the morphological and molecular features of PDO**. A** Representative images of PDO morphology from CRC patients. **B** H&E staining comparing tumor organoids to their matched patient tumor tissue. Scale bar: 50 μm. **C** Representative images of immunofluorescent microscopy about cell type compositions in tumor organoids. Mucin2 (Muc2; red) for goblet cells, Lysozyme (Lysz; green) for paneth cells and Villin (Vill; green) for epithelial cells. Scale bar: 20 μm. **D** Representative images of IHC of CK7, Ki67, CDX2, CK20, HLA-ABC from primary tumor tissue and patient-derived organoids. O: organoids, T: tumor tissue. Scale bar: 50 μm

Tumor samples were obtained from clinical patients with CRC. The clinical characteristics of the patients are outlined in Table 1. Freshly resected CRC tumor tissues were used to establish PDO as previously described [12]. Histological evaluation revealed significant morphological similarities between PDO and the tumor tissue that they were originally-derived. H&E staining illustrated that CRC tumor-derived organoids presented patient-specific heterogeneous morphologies ranging from hollow cystic structures to solid/compact structures (Fig. 1A, B). Multiple types of cells were found within the tumor organoid in a heterogeneous manner, such as paneth cells expressing Lysozyme (Lysz) and epithelial cells expressing Villin 1 (Vill), while a lower abundance of Mucin2 (Muc2)-expressing goblet cells were observed in the tumor organoids (Fig. 1C). An array of immunohistochemistry staining, including caudal type homeobox 2 (CDX2), cytokeratin 7 (CK7), cytokeratin 20 (CK20), Ki67 as well as antigen presenting molecule HLA-ABC, all showed similar staining patterns in the patient tumor tissue and matched PDO as shown in Fig. 1D. In conclusion, these data demonstrated that the PDO recapitulated the histological and genetic features of the original tumor tissues.

**Table 1:** Characteristics of CRC samples

Sample	Sex	Age	Primary tumor/metastasis	Tumor location	Histology	Stage	Degree of differentiation
CRC1	F	80	Primary tumor	Colon	Adenocarcinoma	III	Moderately differentiated
CRC2	M	28	Metastasis	Colon	Adenocarcinoma mixed Signet-ring carcinoma	IV	Poorly differentiated
CRC3	F	67	Metastasis	Colon	Adenocarcinoma	IV	Moderately differentiated
CRC4	M	76	Metastasis	Colon	Adenocarcinoma	IV	Moderately differentiated

### Tumor-specific T cells predominantly exist in TILs

Recent work has shown that tumor-specific T cells can be found within the CD39^+^CD103^+^ subset, PD-1^+^CD8^+^ subset and CXCL13^+^CD8^+^ subset of T cells from patients with epithelial cancers [32–34]. To evaluate the heterogeneity of tumor-specific T cells from CRC1 to CRC4 patients, we analyzed the frequency of CD39^+^CD103^+^, PD-1^+^CD8^+^ subset and CXCL13^+^CD8^+^ subset T cells in peripheral blood lymphocytes (PBLs) and matched tumor-infiltrating lymphocyte (TILs) fragments derived from CRC patients using flow cytometry.

**Fig. 2:**
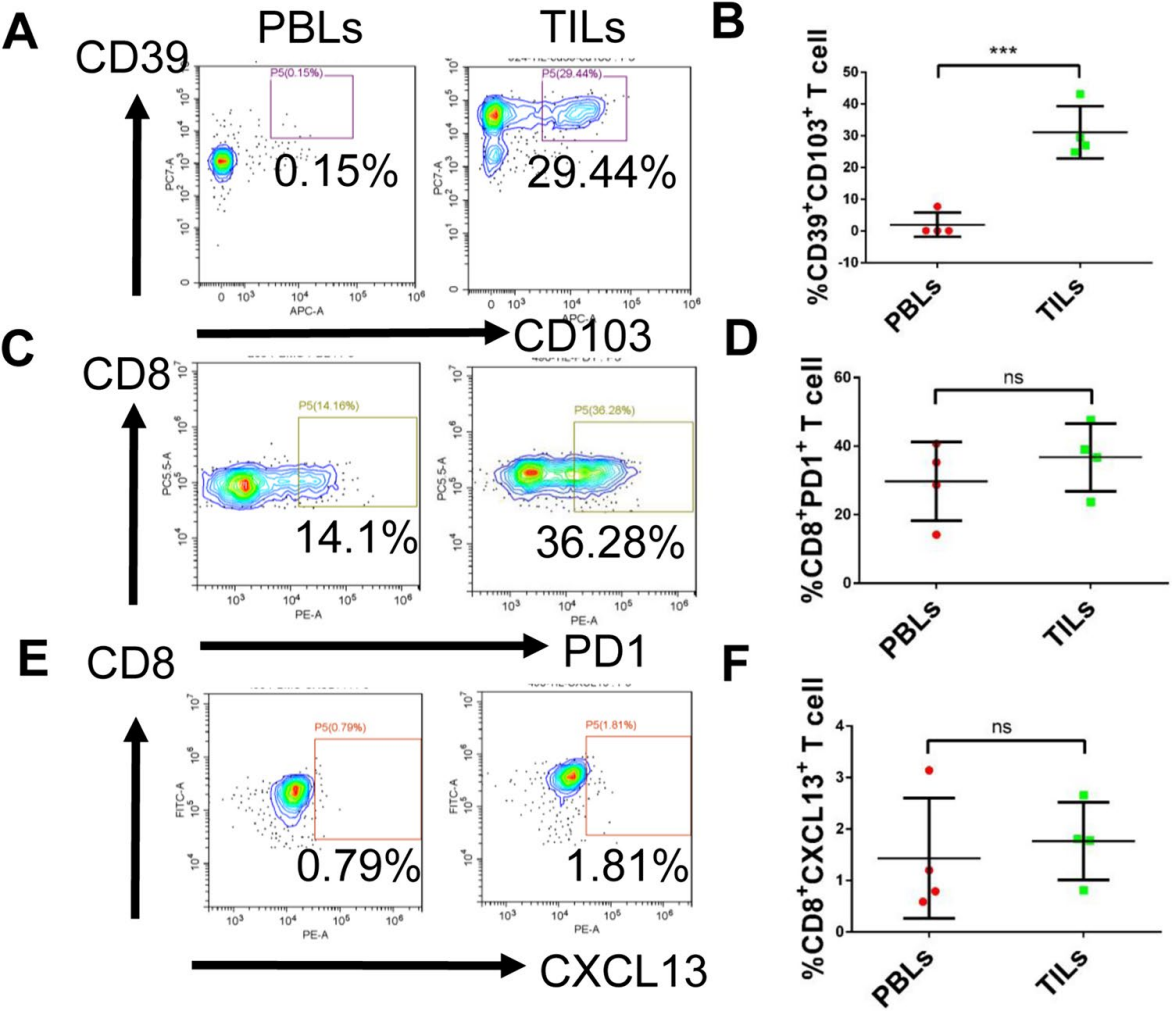
Phenotype analysis of peripheral blood lymphocytes and matched tumor infiltrating lymphocytes form CRC patients. **A** Typical results of flow cytometry analysis revealed the frequency of CD39^+^CD103^+^CD8^+^ T cells in PBLs and matched TILs of CRC patients. **B** Statistical analysis of the frequency of CD39^+^CD103^+^ T cells in PBLs and matched TILs of CRC patients. **C** Typical results of the frequency of CD8^+^PD-1^+^ T cells in PBL and matched TILs of CRC patients. **D** Statistical analysis of the frequency of CD8^+^PD-1^+^ T cells in PBLs and matched TILs of CRC patients. **E** Typical results of the frequency of CD8^+^CXCL13^+^ T cells in PBLs and matched TILs of CRC patients. **F.** Statistical analysis of the frequency of CD8^+^CXCL13^+^ T cells in PBLs and matched TILs of CRC patients

Figure 2A, B show results from CD39^+^CD103^+^CD8^+^ T cells were found in the TILs and PBLs. The frequency of CD39^+^CD103^+^CD8^+^ T cells varied greatly among different CRC patients, with a higher frequency in TILs ranging from 24.9 to 43.1%. However, CD39^+^CD103^+^CD8^+^ T cells were present at a very low frequency in the PBLs, ranging from 0.15 to 7.7%. The frequency of PD-1^+^CD8^+^ T cells and CXCL13^+^ CD8^+^ T cells in TILs fragments ranged from 23.7 to 47.6% and 0.81 to 2.66% respectively. Although no statistically significant difference was observed between PBLs and TILs in these four CRC patients, there was a tendency for a lower frequency in PBLs (Fig. 2C–F). Collectively, these results indicate that tumor-specific T cells predominantly exist in TILs, which shows strong heterogeneity among different patients with CRC.

### Organoid-TIL co-culture to generation of organoid-enriched T cells

**Fig. 3:**
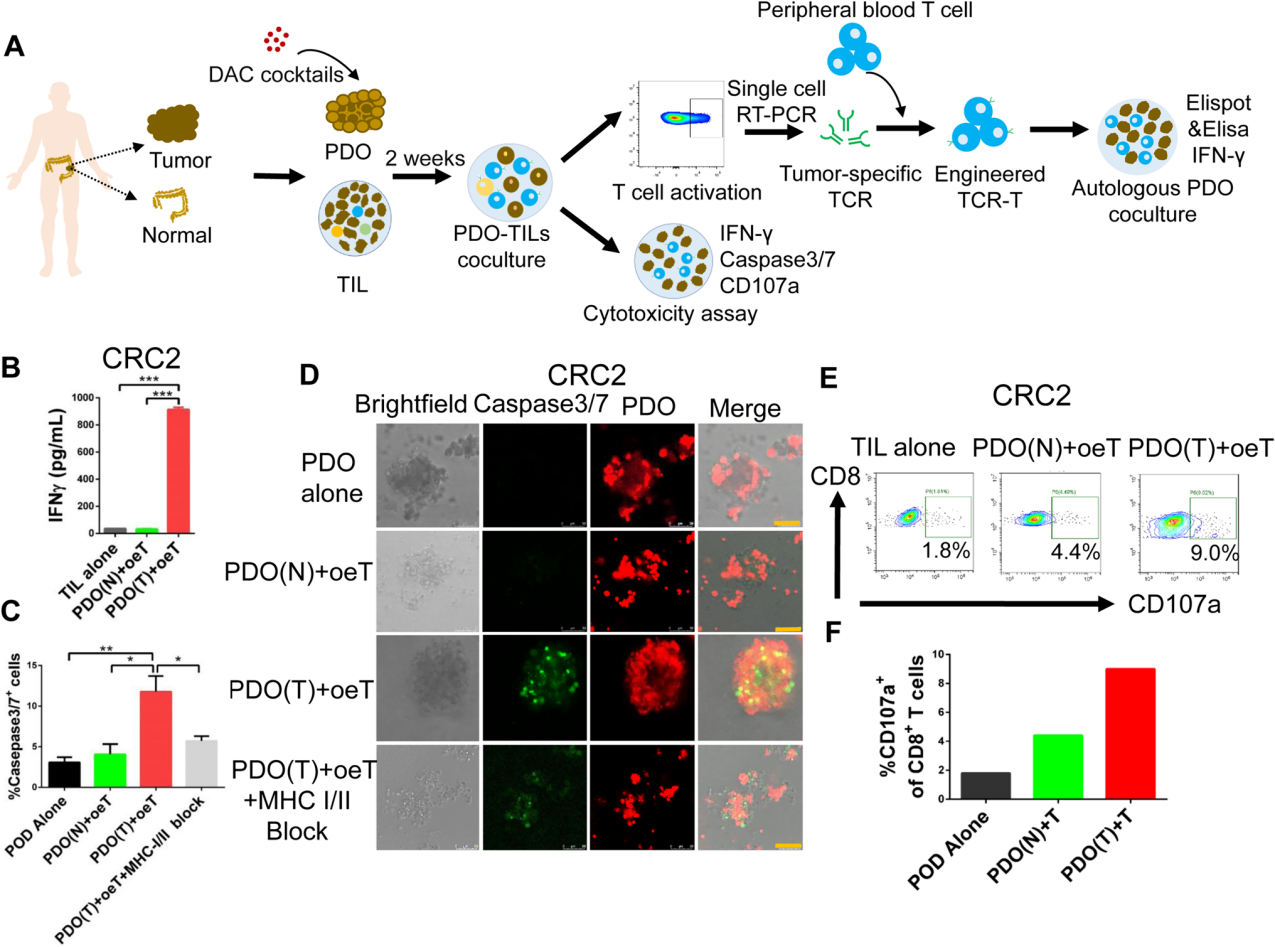
Cytotoxic activity of tumor organoid enriched TILs (oeT). **A** Schematic representation of the experimental workflow. **B** ELISA analysis the IFN-γ secretion by oeT cells after co-cultured with autologous tumor organoids or normal organoids for 24 h. **C** Quantitative summary of the killing efficiency shown by FITC signal (caspase 3/7 probe) in flow cytometry analysis. **D** oeT cells co-cultured with autologous tumor organoids and normal organoids for 20 h to assess killing efficiency respectively. Organoids (red) were labeled with Cell-Trace FarRed, and apoptotic cells (green) were labeled with caspase-3/7 probe. Scale bars: 50 μm. **E** Flow cytometry analyzed oeT cell effector marker CD107a after co-cultured with autologous tumor organoids and normal organoids, respectively. **F** Summary of the frequency of CD107a positive cells after co-cultured with autologous tumor organoids and normal organoids. PDO(N): patient-derived normal organoids, PDO(T): patient-derived tumor organoids

In order to know whether tumor organoids may be used to enrich tumor-specific TILs. We generated PDO and TILs from randomly selected patients with CRC. Prior to co-cultured with autologous TILs, tumor organoids were pre-treatment with DAC cocktails to enhance antigen presentation. TILs were stimulated with autologous tumor organoids for two weeks to obtain oeT cells presented in Fig. 3A.We next assessed whether oeT cells have the ability to respond to autologous tumor PDO. Using an Enzyme-linked immunosorbent (ELISA) assay, oeT cells were stimulated with autologous tumor PDO and normal PDO, respectively. The ELISA outcome showed that oeT cells effectively induced interferon-γ (IFN-γ) secretion when stimulated with tumor PDO, but did not mount a cytotoxic response against normal organoids, demonstrating the tumor specificity of oeT cell-mediated cytotoxic response (Fig. 3B).

In order to determine whether tumor organoids can be effectively destroyed by oeT cells. We separately co-cultured tumor organoids and normal organoids with oeT cells for 20 h and quantified the number of apoptotic tumor cells by flow cytometry. To visualize the cytolytic activity of tumor-specific T cells, we labeled organoids with a tracer dye and imaged them in the presence of a green fluorescent apoptosis probe detecting active caspase-3/7 (‘‘caspase-3/7 probe’’). The results showed that tumor PDO induced more apoptosis than those from the other groups, whereas tumor organoids cultured without oeT cells or with oeT cells in the presence of blocking MHC class I and MHC class II antibodies group induced little apoptosis (Fig. 3C, D, Supplementary Figs. S1 and S2). Furthermore, the frequency of T cell effector markers CD107a was higher in the tumor PDO group (CD107a: 9%) than those from the normal PDO and PDO alone group (CD107a: 4.4%, 1.8%: respectively) (Fig. 3E, F, Supplementary Fig. S3). Taken together, these data demonstrated that oeT cells had better anti-tumor ability.

### Tumor-specific T cells were isolated from co-cultured oeT cells using CD137 expression

**Fig. 4:**
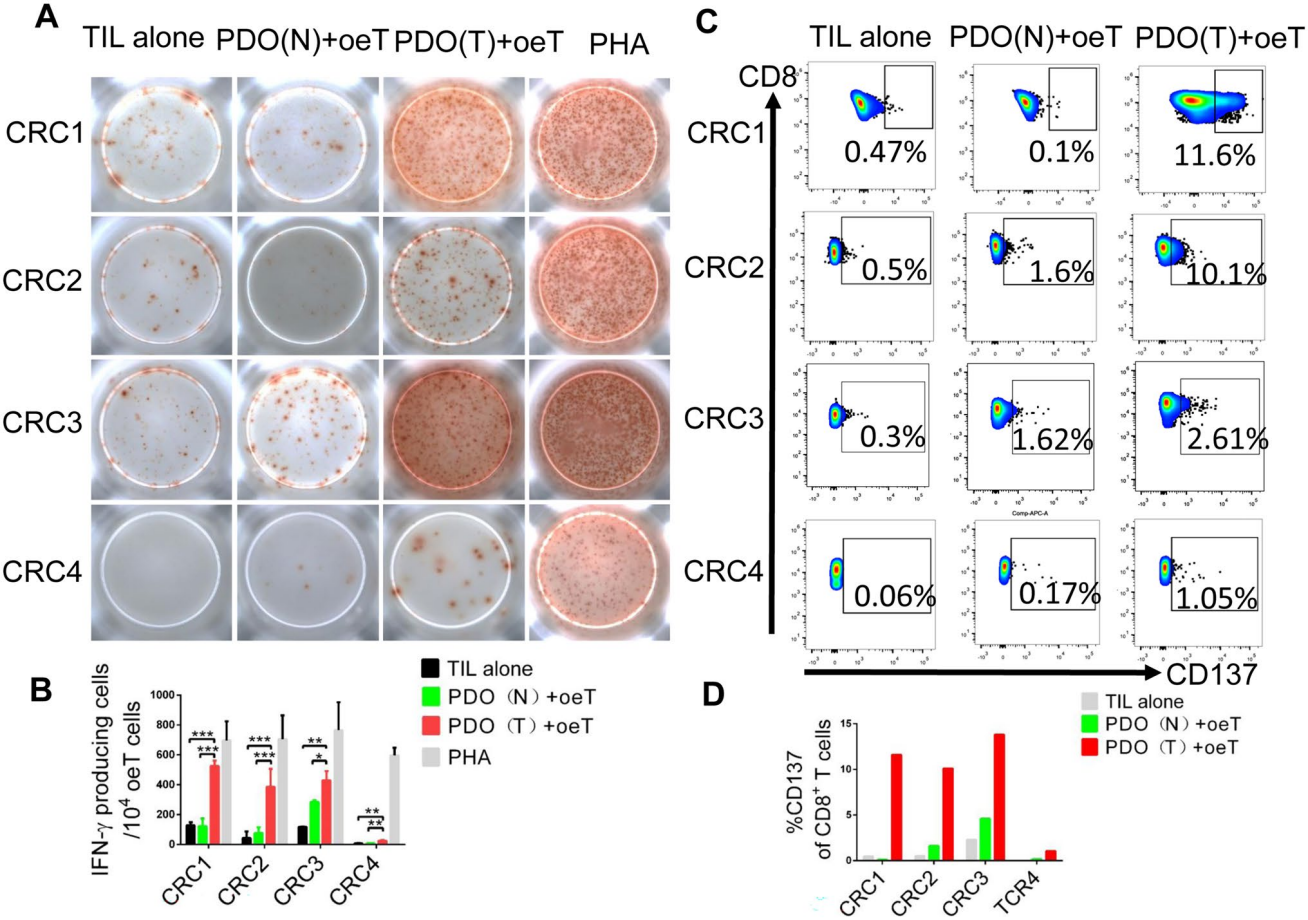
Enrichment of Tumor-specific T cell in TILs by co-culture with autologous tumor organoids. **A** IFN-γ ELISPOT analysis of oeT cells co-cultured with autologous tumor organoids or normal organoids. TILs with no targets are negative controls. PHA well is the positive control. **B** Quantification of organoid-induced IFN-γ production obtained by oeT cells co-cultured with autologous tumor organoids or normal organoids. **C** Representative flow cytometry plots of CD8^+^CD137^+^T cells after oeT cells stimulated with either tumor organoids and normal organoids. **D** Quantification of organoids induced CD137 expression of CD8^+^ T cells directly after oeT cells stimulated with either tumor organoids or normal organoids. PDO (N): patient-derived normal organoids, PDO (T): patient-derived tumor organoids

In order to obtain tumor-specific T cells and their TCRs, oeT cells were stimulated with tumor PDO again, normal PDO and TILs alone as negative controls. T cell responses were assessed through the upregulation of CD137 and secretion of IFN-γ using an enzyme-linked immunospot (ELISPOT) assay. We found that oeT cells co-cultured with tumor PDO exhibited a significantly higher quantity of IFN-γ positive cell spots compared to normal PDO or TIL alone in CRC patients (Fig. 4A, B).

Single CD8^+^ CD137^+^ T cells were isolated and their TCR sequences were amplified using single-cell PCR in 96-well plates.

The tumor-specific TCR may be the most dominant TCR on CD8^+^ CD137^+^ oeT cells. In order to eliminate the possibility of non-specific TCR amplification, oeT cells stimulated with normal PDO and TIL alone were used as a negative control. Meanwhile,

the flow cytometry results showed a significantly higher frequency of tumor-specific T cells in the tumor PDO group compared to both the TIL alone and normal PDO groups among CRC patients. However, little difference in the frequency of tumor-specific T cells between the TIL alone group and normal PDO group (Fig. 4C, D).

### Construction and functional validation of tumor-specific TCR-Ts

**Fig. 5:**
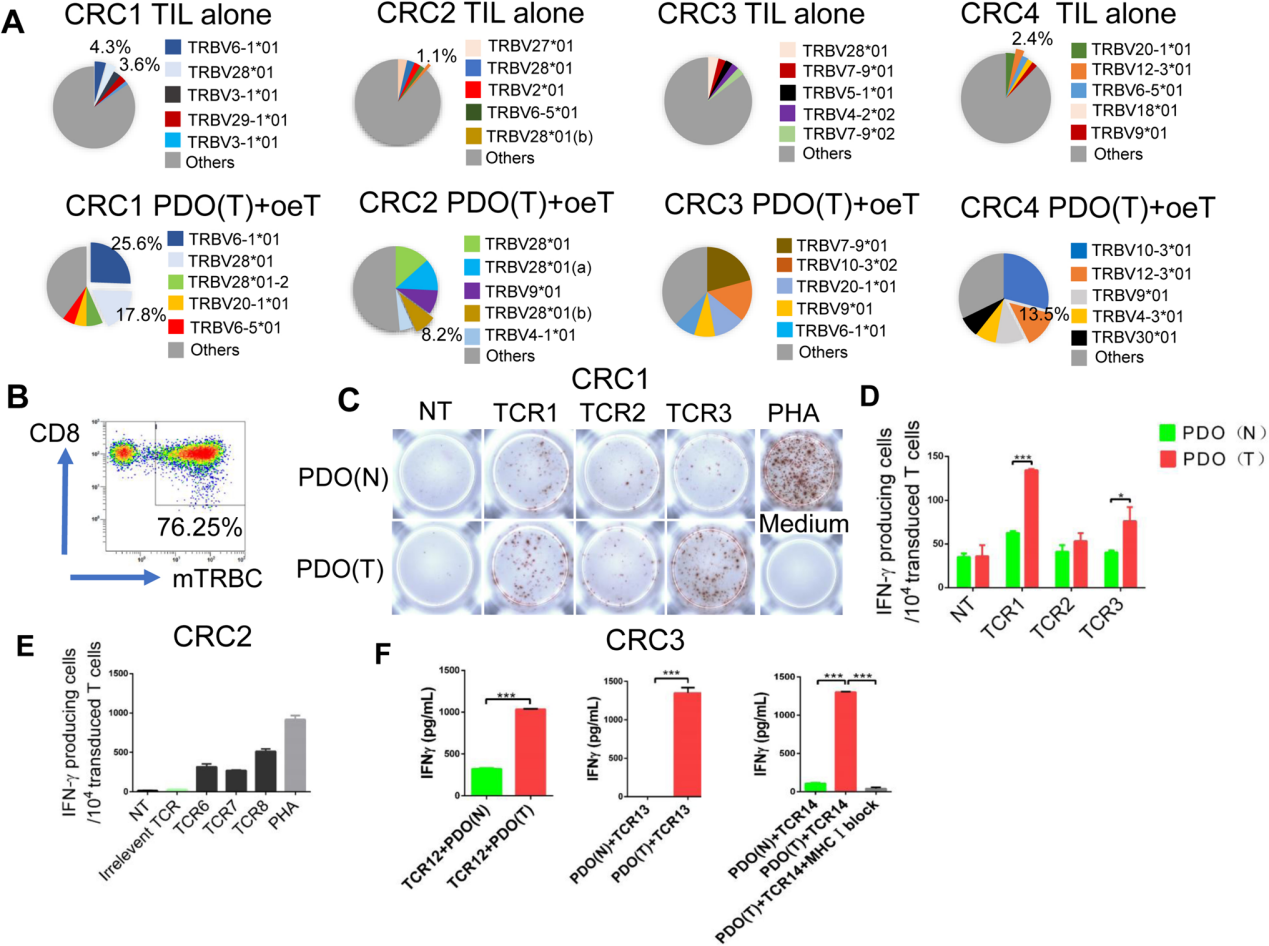
Functional verification of TCR-Ts. **A** Pie charts showing the distribution of a given TCR beta-chains of CD8^+^CD137^+^ T cells stimulated with tumor organoids. **B** PBLs form healthy donors were transduced with the chimeric TCR and flow sorted by anti-murine TCR-β chain constant region antibody. **C** IFN-γ ELISPOT analysis on TCR-T cells that were exposed to autologous tumor organoids or normal organoids in CRC1. NT: no transfection.** D** Quantification of organoid induced IFN-γ production by TCR-T cells co-cultured with autologous tumor organoids or normal organoids in CRC1. **E** Quantification of organoid induced IFN-γ production by TCR-T cells co-cultured with autologous tumor organoids in CRC2. **F** ELISA analysis the IFN-γ secretion by TCR-T cells after co-cultured with autologous tumor organoids or normal organoids for 24 h in CRC3. PDO (N): patient-derived normal organoids, PDO (T): patient-derived tumor organoids

To determine if oeT cells are clonally proliferated subsets of tumor-specific T cells, we isolated CD8^+^ CD137^+^ T cells from both the TIL alone group and the oeT cells from the tumor PDO-stimulated group. The TCR α/β chain pairs were then identified using single-cell RT-TCR sequencing. The results showed that no TCR was represented more than 4.3% of the TIL alone group in CRC patients, demonstrating the polyclonal biological characteristics of the subpopulations (Fig. 5A). However, the TCRs of tumor PDO group showed significant oligoclonal. In CRC1, oeT cells were predominantly composed of TCR1 and TCR2, accounting for 25.6% and 17.8% of the T cell populations, respectively. In contrast, the TIL alone group had a much lower proportion of TCR1 and TCR2, representing only 4.3% and 3.6% of these subsets. In CRC2, the frequency of TCR9 was 1.1% in TIL alone and increased to 8.2% in oeT cells, indicating a more than 7-folds clonal expansion after incubation with autologous tumor PDO in vitro. In CRC3, the TCR11 was undetectable among the top five in TIL alone, but it was enriched to 11.4% in oeT cells, demonstrating a more than 28-folds clonal expansion (Table 2). In CRC4, TCR17 underwent more than a 5-folds clonal proliferation compared to before stimulation, increasing from 2.4% in TIL alone to 13.5% in oeT cells after co-culture with autologous tumors (Fig. 5A).

**Table 2:** CDR3 sequences and their frequencies of the top5 TCRs from CRC patients

TCR	TRAV	TRAJ	TRA-CDR3	TRBV	TRBD	TRBJ	TRB-CDR3	TIL alone (Freq%)	PDO(T)-oeT (Freq%)
CRC1									
TCR1	TRAV35*02	TRAJ10*01	AGVTGGGNKLT	TRBV6-1*01	TRBD1*01	TRBJ1-2*01	ASSEGTGFYGYT	4.3	25.6
TCR2	TRAV35*02	TRAJ 10*01	AGVTGGGNKLT	TRBV28*01	TRBD2*01	TRBJ2-2*01	ASSLTSASGELF	3.6	17.8
TCR3	TRAV24*01	TRAJ12*01	APWMDSSYKLI	TRBV28*01	TRBD1*01	TRBJ1-2*01	ASSIGGHDGYT	2.5	6.7
TCR4	TRAV8-3*02	TRAJ15*01	AVGAQAGRNCSD	TRBV20-1*01	TRBD1*01	TRBJ2-7*01	SASPQSYEQY	1.2	5
TCR5	TRAV36/DV7*04	TRAJ30*01	AVSRDDKII	TRBV6-5*01	TRBD2*01	TRBJ2-2*01	ASSYVGNTGELF	1.2	5
CRC2									
TCR6	TRAV8-3*01	TRAJ21*01	AVGDNFNKFY	TRBV28*01	TRBD1*01	TRBJ2-2*01	ASSYVGNTGELF	1.1	13.4
TCR7	TRAV14/DV4*03	TRAJ24*03	AMREGADSWGKFQ	TRBV28*01	TRBD1*01	TRBJ1-4*01	ASSLHGRGDEKLF	0.5	12.4
TCR8	TRAV23/DV6*01	TRAJ45*01	ALKAGGGASKLT	TRBV9*01	TRBD2*02	TRBJ2-1*01	ASSVGGGVDEQF	2.8	9.3
TCR9	TRAV1-2*01	TRAJ6*01	AVRGTLSGGSYIPT	TRBV28*01	TRBD1*01	TRBJ2-2*01	ASSATSASGELF	1.1	8.2
TCR10	TRAV13-1*01	TRAJ42*01	AASPPGGSQGNLI	TRBV4-1*01	TRBD2*01	TRBJ2-1*01	ASSHRTSSRGEQF	0.5	3.1
CRC3									
TCR11	TRAJ27*01	TRAJ27*01	AVSGVGKST	TRBV7-9*01	TRBD1*01	TRBJ2-1*01	ASSPPQGATYNEQF	0.4	11.4
TCR12	TRAV10*01	TRAJ16*02	VSGFSDGQKLL	TRBV10-3*02	TRBD1*01	TRBJ2-1*01	ATTKTGERPDEQF	1.3	8
TCR13	TRAV14/DV4*01	TRAJ45*01	ANDSGGGADGLT	TRBV20-1*01	TRBD2*02	TRBJ2-1*01	SARGLLGGRAREQF	3	4
TCR14	TRAV10*01	TRAJ4*01	VVSFSGGYNKLI	TRBV9*01	TRBJ1-2*01	TRBD1*01	ASSVGDRDYGYT	0	4
TCR15	TRAV8-6*02	TRAJ20*01	AVSGRSNDYKLS	TRBV6-1*01	TRBD1*01	TRBJ1-3*01	ASSEKRQGAGNTIY	0.4	3
CRC4									
TCR16	TRAV8-2*01	TRAJ43*01	AVSDLGNDMR	TRBV10-3*01	TRBD2*01	TRBJ2-3*01	AISGGAGATDTQY	4	31
TCR17	TRAV20*01	TRAJ4*01	AVFSGGYNKLI	TRBV12-3*01	TRBD2*01	TRBJ1-5*01	ASSLEFYSNQPQH	2.4	13.5
TCR18	TRAV3*01	TRAJ31*01	AVRDEGARLM	TRBV9*01	TRBD1*01	TRBJ2-3*01	ASSIFSGTGGADTQY	0	10.3
TCR19	TRAV22*01	TRAJ26*01	AVGRGYGQNFV	TRBV4-3*01	TRBD1*01	TRBJ1-4*01	ASSQDGGLNEKLF	0	7.7
TCR20	TRAV13-2*01	TRAJ8*01	AENMGFQKLV	TRBV30*01	TRBD1*01	TRBJ1-2*01	AWTQGANGYT	0	7.1

We sequenced the Vβ and Vα chains of TCRs in oeT cells and chose the top five to create chimeric TCRs (Table 2). For enhanced expression of introduced TCRs in T cells, the TCRs were designed in a β/α chain sequence and the constant regions were replaced with mouse-derived counterparts that had been altered to include interchain disulfide bonds and hydrophobic modifications, as previously documented [35]. The TCRs were lentivirus-transduced into healthy donor T cells to generate TCR-Ts. Then these T cells were sorted and achieved a positive rate of more than 76.25%, as shown in Fig. 5B. We then investigated whether these TCR-Ts could specifically identify and mediate effector functions in response to autologous tumor PDO through releasing cytokines. Engineered TCR1-T cells and TCR3-T cells exhibited increased levels of IFN-γ secretion and specific cytotoxicity toward autologous tumor PDO compared to normal PDO (Fig. 5C, D). In CRC2, three out of the five selected TCRs could specifically recognize autologous tumor cells, as evidenced by the IFN-γ production of these engineered TCR-T cells when incubated with autologous tumor cells (Fig. 5E, supplementary Fig. S4). Co-culture of the engineered TCR12-Ts and TCR13-Ts cells with autologous tumor organoids induced significant IFN-γ secretion. While incubating them with normal organoids did not induce detectable IFN-γ secretion. Additionally, we revealed that that TCR14-Ts displayed significantly inhibited IFN-γ secretion levels with MHC class I blocking antibody, indicating that the tumor recognition by TCRs is highly MHC-dependent (Fig. 5F). These findings provided direct evidence that the ability of the TCRs identified in oeT cells were sufficient to confer the tumor-targeting ability to engineer TCR-T cells.

## Discussion

Although previous studies have documented the sustained regression of metastatic colorectal cancer and metastatic melanoma through the adoptive transfer of in vitro expanded autologous TILs, the efficacy of this therapy is limited in numerous solid tumors due to the presence of a large number of bystander T cells and exhausted cell phenotypes [36–38]. Therefore, efficiently identifying tumor-specific TCRs and introduced into peripheral T cells to generate tumor-specific TCR-Ts is an optimal strategy for clinical therapy. In this study, we developed a co-culturing platform for the enrichment and expansion of tumor-specific TILs in vitro through repeated stimulation of autologous tumor organoids from CRC patients. The identification of tumor-specific TCRs by flow sorting of CD8^+^CD137^+^ T cells and the TCRs subsequent introduction of PBLs showed specific tumor reactivity and cytotoxicity. The organoid-autologous TILs co-culture is an effective system for identifying tumor-specific TCRs for TCR-T therapy. Further experiments will need to assess the effectiveness of tumor-specific TCRs for adoptive cell therapy.

Previous studies have reported the generation of tumor-specific T cells by co-incubating tumor organoids with peripheral blood [39]. However, this method can be overall low-yield, possibly due to the low frequency of tumor-specific T cells in peripheral blood. In this study, we systematically analyzed the frequency of tumor-specific T cells in peripheral blood and matched TILs from the same patient. we found that the frequency of CD39^+^CD103^+^ T cells in the TILs was much higher than in the matched peripheral blood from the same patient, which suggests that TILs have a higher frequency of tumor-specific T cells compared to those found in peripheral blood. The consistent results with previous reports, neoantigen-specific T cells that are occurring multiple times in TILs and found at a frequency greater than twice the limit of detection in the peripheral blood [20, 32]. Hence, the use of TILs, which greatly enrich tumor-specific T cells, can improve the efficiency of identifying tumor-specific TCRs through organoid stimulation.

Previous studies have focused on screening tumor-specific TCRs using predicted tumor antigens [5, 40]. However, there is less evidence about screening tumor-specific TCRs using natural antigens from tumor organoids. Therefore, in terms of whether tumor-specific TCRs could be enriched by tumor organoids-autologous TILs immune co-cultures, we found that the proportion of CD137-positive T cells after tumor organoids co-cultures was significantly higher than the control group. Meanwhile, we demonstrated a high degree of clonal expansion of TCRs from CD8^+^ oeT after sequencing analysis. These results showed that tumor-specific TCRs can be obtained by co-culture of autologous tumor organoids with TILs in vitro. And their TCRs can adequately recognized autologous tumor organoids in a MHC-dependent manner. Previous studies have demonstrated that tumor organoids can accurately reflect the mutanome of their parental tumor tissue, as well as the diversity in MHC class I peptide presentation, including tumor antigens [16, 41]. Combined with these results, we showed that tumor organoids can be perform as natural tumor antigens for enrichment of tumor-specific TCRs.

There are some limitations in this study. Firstly, previous study showed that tumor organoids express MHC class II proteins in pancreatic cancer, suggesting that the tumor cells present MHC class II antigens for CD4^+^ T cells [42]. Our study mainly focuses on tumor-specific CD8^+^ T cells and their TCRs specific response and cytotoxicity to autologous tumor cells after organoid enrichment. Therefore, we plan to investigate whether colorectal organoids co-cultured with TILs can effectively enrich CD4^+^ tumor-specific T cells in the future. In addition, we identified tumor-specific TCRs using low-throughput single-cell RT-PCR and Sanger sequencing. This approach is limited in comprehending the comprehensive information of TCR repertoire after stimulation with organoid. High-throughput single-cell transcriptome sequencing will be incorporated into future studies, which would provide us with a more complete picture of the genetic composition of tumor-specific TCRs. Finally, in this study using tumor organoids as the source of natural tumor antigen to screen tumor-specific TCRs, however, the specific antigen epitope recognized by tumor-specific TCRs remains unknown. A variety of approaches have been used to address this issue, including T cell trogocytosis and baculoviral peptide libraries [43, 44]. These methods provide valuable insights for the identification of specific antigen epitopes after known cognate tumor-specific TCRs.

### Supplementary Information

Below is the link to the electronic supplementary material.

Supplementary file1 (PDF 785 kb)

## Data Availability

The data generated in this study are available within the article and its supplementary data files.
