# Supplementary material for: Identification and validation of tumor-specific T cell receptors from tumor infiltrating lymphocytes using tumor organoid co-cultures
Source: Cancer Immunol Immunother. 2024 Jul 2;73(9):164. doi: 10.1007/s00262-024-03749-8 (PMC11219989; doi:10.1007/s00262-024-03749-8)
Supplement: Supplementary file 1 — Supplementary file1 (PDF 785 kb) [file 262_2024_3749_MOESM1_ESM.pdf]

Supplementary Table S1.Antibodies (immunofluorescence/immunohistochemical)

Antibody	Catalog Number	Source	Dilution
MUC2	sc7314	santa cruz	1:200
Lysozyme	ab108508	Abcam	1:200
Villin-1 (R814)	2369s	Cell signaling technology	1:200
Cytokeratin7	ab181598	Abcam	1:8000
Ki67	ab15580	Abcam	1:500
CDX2	ab15481	Abcam	1:200
CK20	ab181598	Abcam	1:200
HLA-ABC	ab225636	Abcam	1:200
Anti-rabbit secondary Ab	A21206	Life technologies	1:500
Anti-mouse secondary Ab	ZF-0512	ZSGB-BIO	1:300

Supplementary Table S2. Antibodies of flow cytometry

Antibody	Catalog Number	Source	Clone(RRID)
anti-human CD3 (FITC-conjugated)	317306	BioLegend	OKT3 (AB_571907)
anti-human CD8 (PE-conjugated)	344706	BioLegend	SK1 (AB_1953244)
anti-human CD137 (APC-conjugated)	309810	BioLegend	4B4-1(AB_830672)
anti-human CD107a (PE-Cyanine7-conjugated)	328618	BioLegend	H4A3(AB_11147955)
7-AAD Viability Staining Solution	420403	BioLegend	
anti-human CD39(Percp/cyanine5.5-conjugated)	328218	Biolegend	A1(AB_2562897)
anti-human CD103(APC-conjugated)	350216	Biolegend	Ber-ACT8(AB_2563907)
anti-human PD-1 (PE-conjugated)	379210	Biolegend	A17188A(AB_2922607)
anti-human CXCL13 Antibody (PE-conjugated)	MA5-23666	invitrogen	53610(AB_2610226)
anti-human CD8 (PerCP/Cyanine5.5-conjugated)	980918	Biolegend	SK1
anti-human CD8 (FITC-conjugated)	344703	Biolegend	SK1 (AB_1877179)
Pan-MHC Class I antibody	311428	Biolegend	W6/32(AB_2561492)
anti-human HLA-DR/DP/DQ	555556	BD	Tu39
TCR beta Monoclonal Antibody (APC-conjugated)	17-5961-82	eBioscience	H57-597
Pan-anti-human CD279 (PD-1) Antibody	329926	Biolegend	EH12.2H7(AB_11147365)

Supplementary Table S3. Single-cell RT-PCR primers

TRA	1st round PCR primers（EXT)	2nd round PCR primers（INT)
TRAV1	AACTGCACGTACCAGACATC	GCACCCACATTTCTKTCTTAC
TRAV2	CACTCTGTGTCCAATGCTTAC	GATGTGCACCAAGACTCC
TRAV3	AAGATCAGGTCAACGTTGC	ATGCACCTATTCAGTCTCTGG
TRAV4	CTCCATGGACTCATATGAAGG	ATTATATCACGTGGTACCAACAG
TRAV5	CTTTTCCTGAGTGTCCGAG	TACACAGACAGCTCCTCCAC
TRAV6	CACCCTGACCTGCAACTATAC	TGGTACCGACAAGATCCAG
TRAV7	AGCTGCACGTACTCTGTCAG	ACAATTTGCAGTGGTACAGG
TRAV8-1	CTCACTGGAGTTGGGATG	GTCAACACCTTCAGCTTCTC
TRAV8-2,8-4	GCCACCCTGGTTAAAGG	AGAGTGAAACCTCCTTCCAC
TRAV8-3	CACTGTCTCTGAAGGAGCC	TTTGAGGCTGAATTTAAGAGG

TRAV8-6	GAGCTGAGGTGCAACTACTC	AACCAAGGACTCCAGCTTC
TRAV8-7	CTAACAAAGGCCACCCAG	ATCAGAGGTTTTGAGGCTG
TRAV9-1,9-2	TGGTATGTCCAATATCCTGG	GAAACCACTTCTTTCCACTTG
TRAV10	CAAGTGGAGCAGAGTCCTC	GAAAGAACTGCACTCTTCAATG
TRAV12-1,12-2,12-3	CARTGTTCCAGAGGGAGC	AAGATGGAAGGTTTACAGCAC
TRAV13-1	CATCCTTCAACCCTGAGTG	TCAGACAGTGCCTCAAACTAC
TRAV13-2	CAGCGCCTCAGACTACTTC	CAGTGAAACATCTCTCTCTGC
TRAV14	AAGATAACTCAAACCCAACCAG	AGGCTGTGACTCTGGACTG
TRAV16	AGTGGAGCTGAAGTGCAAC	GTCCAGTACTCCAGACAACG
TRAV17	GGAGAAGAGGATCCTCAGG	CCACCATGAACTGCAGTTAC
TRAV18	TCCAGTATCTAAACAAAGAGCC	TGACAGTTCCTTCCACCTG
TRAV19	AGGTAACTCAAGCGCAGAC	TGTGACCTTGGACTGTGTG
TRAV20	CACAGTCAGCGGTTTAAGAG	TCTGGTATAGGCAAGATCCTG
TRAV21	TTCCTGCAGCTCTGAGTG	AACTTGGTTCTCAACTGCAG
TRAV22	GTCCTCCAGACCTGATTCTC	CTGACTCTGTGAACAATTTGC
TRAV23	TGCTTATGAGAACACTGCG	TGCATTATTGATAGCCATACG
TRAV24	CTCAGTCACTGCATGTTCAG	TGCCTTACACTGGTACAGATG
TRAV25	GGACTTCACCACGTACTGC	TATAAGCAAAGGCCTGGTG
TRAV26-1	GCAAACCTGCCTTGTAATC	CGACAGATTCACTCCCAG
TRAV26-2	AGCCAAATTCAATGGAGAG	TTCACTTGCCTTGTAACCAC
TRAV27	TCAGTTTCTAAGCATCCAAGAG	CTCACTGTGTACTGCAACTCC
TRAV29	GCAAGTTAAGCAAAATTCACC	CTGCTGAAGGTCCTACATTC
TRAV30	CAACAACCAGTGCAGAGTC	AGAAGCATGGTGAAGCAC
TRAV34	AGAACTGGAGCAGAGTCCTC	ATCTCACCATAAACTGCACG
TRAV35	GGTCAACAGCTGAATCAGAG	ACCTGGCTATGGTACAAGC
TRAV36	GAAGACAAGGTGGTACAAAGC	ATCTCTGGTTGTCCACGAG
TRAV38-1,38-2	GCACATATGACACCAGTGAG	CAGCAGGCAGATGATTCTC
TRAV39	CTGTTCCTGAGCATGCAG	TCAACCACTTCAGACAGACTG
TRAV40	GCATCTGTGACTATGAACTGC	GGAGGCGGAAATATTAAAGAC
TRAV41	AATGAAGTGGAGCAGAGTCC	TTGTTTATGCTGAGCTCAGG
TRAC	GACCAGCTTGACATCACAG	TGTTGCTCTTGAAGTCCATAG

TRB	1st round PCR primers (EXT)	2st round PCR primers （INT）
TRBV2	TCGATGATCAATTCTCAGTTG	TTCACTCTGAAGATCCGGTC
TRBV3-1	CAAAATACCTGGTCACACAG	AATCTTCACATCAATTCCCTG
TRBV4-1, 4-2, 4-3	TCGCTTCTCACCTGAATG	CCTGCAGCCAGAAGACTC
TRBV5-1, 5-3, 5-4	GATTCTCAGGKCKCCAGTTC	CTTGGAGCTGGRSGACTC
TRBV5-5, 5-6, 5-7, 5-8	GTACCAACAGGYCCTGGGT	TCTGAGCTGAATGTGAACG
TRBV6-1, 6-2, 6-3, 6-5,	ACTCAGACCCCAAAATTCC	GTGTRCCCAGGATATGAACC

6-6, 6-7, 6-8, 6-9		
TRBV6-4	ACTGGCAAAGGAGAAGTCC	TGGTTATAGTGTCTCCAGAGC
TRBV7-1, 7-2, 7-3	TRTGATCCAATTTCAGGTCA	TCYACTCTGAMGWTCCAGCG
TRBV7-4,7-6,7-7,7-8,7-9	GSWTCTYTGCAGARAGGCC	TGRMGATYCAGCGCACA
TRBV9	GATCACAGCAACTGGACAG	GTACCAACAGAGCCTGGAC
TRBV10-1, 10-2, 10-3	TGTWCTGGTATCGACAAGACC	TCCYCCTCACTCTGGAGTC
TRBV11-1, 11-2, 11-3	CGATTTTCTGCAGAGACGC	GACTCCACTCTCAAGATCCA
TRBV12-3, 12-4, 12-5	ARGTGACAGARATGGGACAA	CYACTCTGARGATCCAGCC
TRBV13	AGCGATAAAGGAAGCATCC	CATTCTGAACTGAACATGAGC
TRBV14	CCAACAATCGATTCTTAGCTG	ATTCTACTCTGAAGGTGCAGC
TRBV15	AGTGACCCTGAGTTGTTCTC	ATAACTTCCAATCCAGGAGG
TRBV16	GTCTTTGATGAAACAGGTATGC	GAAAGATTTTCAGCTAAGTGCC
TRBV17	CAGACCCCCAGACACAAG	TGTTCACTGGTACCGACAG
TRBV18	CATAGATGAGTCAGGAATGCC	CGATTTTCTGCTGAATTTCC
TRBV19	AGTTGTGAACAGAATTTGAACC	TTCCTCTCACTGTGACATCG
TRBV20-1	AAGTTTCTCATCAACCATGC	ACTCTGACAGTGACCAGTGC
TRBV23-1	GCGATTCTCATCTCAATGC	GCAATCCTGTCCTCAGAAC
TRBV24-1	CCTACGGTTGATCTATTACTCC	GATGGATACAGTGTCTCTCGA
TRBV25-1	ACTACACCTCATCCACTATTCC	CAGAGAAGGGAGATCTTTCC
TRBV27, 28	TGGTATCGACAAGACCCAG	TTCYCCCTGATYCTGGAGTC
TRBV29-1	TTCTGGTACCGTCAGCAAC	TCTGACTGTGAGCAACATGAG
TRBV30	TCCAGCTGCTCTTCTACTCC	AGAATCTCTCAGCCTCCAGAC
TRBC	AGGCAGTATCTGGAGTCATT	ACTGTGCACCTCCTTCCCAT

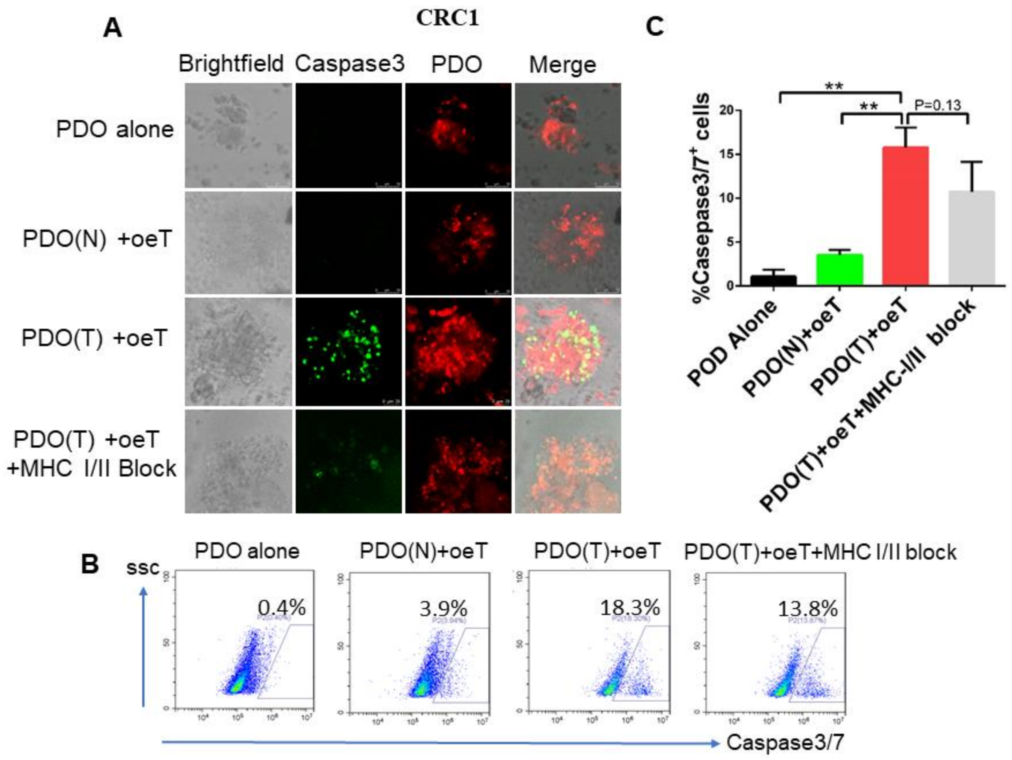

Supplementary Fig.S1. A. oeT cells co-cultured with autologous tumor organoids and normal PDO for 20 hours to assess killing efficiency respectively. Organoids (red) were labelled with Cell-Trace FarRed, and apoptotic cells (green) were labelled with caspase-3/7 probe. Scale bars; 50 μm. **B**. Flow cytometry analysis of the killing efficiency based on FITC signal (caspase 3/7 probe). **C**. Quantitative summary of the frequency of caspase3/7 positive cells. PDO(N): patient-derived normal organoids, PDO(T): patient-derived tumor organoids.

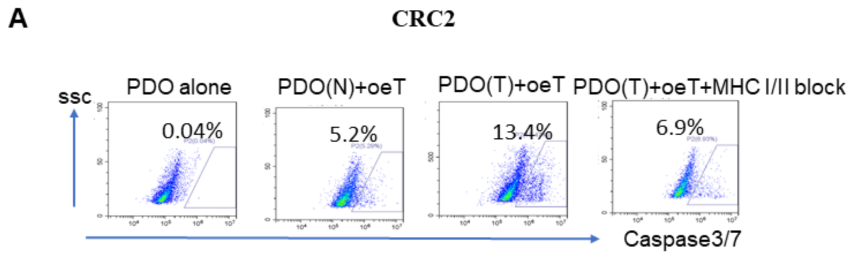

Supplementary Fig.S2.

A. Flow cytometry analysis of the killing efficiency using FITC signal (caspase 3/7 probe) in CRC2. PDO (N): patient-derived normal organoids, PDO (T): patient-derived tumor organoids.

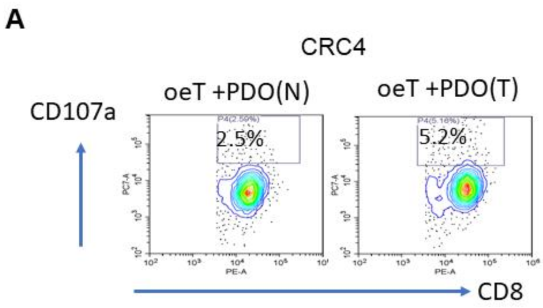

Supplementary Fig.S3.

A. Representative flow cytometry plots of $CD8^{+}CD107a^{+}$ T cells after oeT cells stimulated with either tumor organoids or normal organoids in CRC4. PDO (N): patient-derived normal organoids, PDO (T): patient-derived tumor organoids.

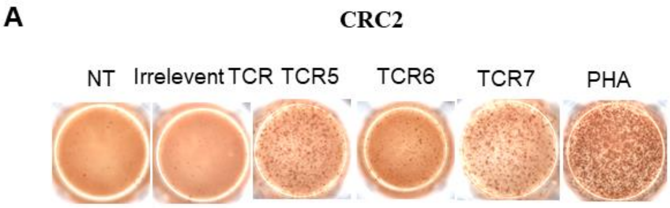

Supplementary Fig.S4.

A. IFN-γ ELISPOT analyzed TCR-T cells were exposed to autologous tumor organoids or normal organoids in CRC2. NT: no transfection.
